# Nonlinear Association Between the C-Reactive Protein–Triglyceride–Glucose Index and Rheumatoid Arthritis Risk: The Mediating Role of Body Mass Index

**DOI:** 10.1155/mi/8729780

**Published:** 2025-11-10

**Authors:** Haiping Xie, Qinwen Liu, Xuefeng Xu, Yanfang Wu, Jianwen Liu, DianTian Lin, Meng Zhou, Zhihan Chen, Fei Gao, Liangchun Cai

**Affiliations:** ^1^Department of Rheumatology and Immunology, Shengli Clinical Medical College of Fujian Medical University, Fujian Provincial Hospital, Fuzhou University Affiliated Provincial Hospital, Fuzhou 350001, China; ^2^Department of Endocrinology, Shengli Clinical Medical College of Fujian Medical University, Fujian Provincial Hospital, Fuzhou University Affiliated Provincial Hospital, Fuzhou 350001, China; ^3^Department of Gastroenterology, Shengli Clinical Medical College of Fujian Medical University, Fujian Provincial Hospital, Fuzhou University Affiliated Provincial Hospital, Fuzhou 350001, China

**Keywords:** body mass index, C-reactive protein-triglyceride-glucose index, mediation analysis, NHANES, rheumatoid arthritis

## Abstract

**Background:**

Rheumatoid arthritis (RA) is a growing public health concern with rising incidence worldwide. The C-reactive protein–triglyceride–glucose index (CTI), a composite marker of inflammation and insulin resistance, has been linked to various metabolic disorders, but its role in RA remains unclear. This study aimed to examine the association between CTI and RA risk and assess whether body mass index (BMI) mediates this relationship.

**Methods:**

We analyzed data from 4292 participants using the 2005–2010 National Health and Nutrition Examination Survey (NHANES). CTI was computed and stratified into quartiles. Multivariable logistic regression models assessed the association between CTI and RA after adjusting for demographic, socioeconomic, lifestyle, and clinical confounders. Restricted cubic spline (RCS) functions were employed to test for nonlinear patterns. Additionally, subgroup analyses examined effect modification, and mediation analysis quantified the indirect effect through BMI.

**Results:**

Elevated CTI values were independently linked to higher odds of RA. After full adjustment, each one-unit rise in CTI corresponded to a 45% increase in RA odds (OR = 1.45, 95% CI: 1.22–1.73, *p*  < 0.001). The RCS analysis demonstrated a significant nonlinear association (p for nonlinearity = 0.048). Stratified analyses indicated consistent patterns across sex, ethnicity, and other variables, with a more pronounced effect among individuals without diabetes (p for interaction = 0.036). Mediation findings showed that BMI accounted for 32.31% of the total CTI–RA effect (*p*  < 0.001).

**Conclusions:**

CTI is nonlinearly and independently associated with RA risk, partly through BMI, highlighting its potential as a biomarker linking metabolic and inflammatory pathways.

## 1. Introduction

Rheumatoid arthritis (RA) is a chronic, systemic autoimmune condition characterized by persistent inflammation of the synovial joints, progressive articular damage, and heightened risks of disability and systemic complications, particularly cardiovascular events [1]. Globally, RA impacts approximately 0.5%–1% of the adult population, with a disproportionate prevalence among women and the elderly [2]. Recent epidemiological data indicate a continuing increase in RA incidence worldwide, attributable not only to population aging but also to modifiable lifestyle factors such as excess adiposity and metabolic disturbances [3]. While traditional studies have predominantly centered on genetic predisposition and autoimmune mechanisms in RA development, emerging research highlights the contribution of systemic low-grade inflammation and metabolic dysregulation to disease onset [4]. Within this framework, integrative biomarkers that reflect both inflammatory and metabolic burden are gaining attention as potential early indicators of RA risk.

The C-reactive protein–triglyceride–glucose index (CTI) is an emerging composite marker that reflects both systemic inflammation and insulin resistance. It has been investigated in the context of several chronic conditions, including cardiovascular disorders, cerebrovascular events, and cancer outcomes [5]. Prior research has demonstrated that elevated CTI levels are linked to increased risks of stroke associated with hypertension [5] as well as depressive symptoms [6]. However, its relevance in the context of autoimmune diseases, particularly RA, has yet to be thoroughly examined. Notably, each of CTI's individual components—C-reactive protein (CRP), fasting glucose, and serum triglycerides—has been independently associated with inflammatory joint conditions [7, 8]. Additionally, findings from recent analyses based on the National Health and Nutrition Examination Survey (NHANES) have revealed that composite indicators reflecting diet-related inflammation, such as the dietary antioxidant index, correlate significantly with RA occurrence. These studies also suggest that body mass index (BMI) acts as a partial mediator in such associations [3]. Taken together, this raises the possibility that BMI may similarly mediate the link between CTI and RA risk.

BMI serves as a fundamental indicator of adiposity and plays a critical role as an immunometabolic biomarker. Beyond its role in energy storage, adipose tissue functions as a hormonally active organ, releasing adipokines—such as leptin and resistin—as well as pro-inflammatory cytokines including tumor necrosis factor-α and interleukin-6, which are implicated in the pathogenesis of RA [9, 10]. Evidence from meta-analyses indicates that obesity elevates RA risk by approximately 30%, independently of genetic predisposition or serologic status [11]. Moreover, excess body weight is linked to poorer disease outcomes and diminished efficacy of biologic therapies in RA patients [12, 13]. Previous mediation analyses have demonstrated that BMI partly accounts for associations between dietary inflammation scores and RA or related traits [4, 14]. These observations support the hypothesis that BMI may function as a mediator in the pathway connecting CTI and RA, reflecting a shared axis of inflammation, metabolism, and autoimmunity. Accordingly, this study aims to examine the association between CTI and RA risk among U.S. adults and to evaluate whether BMI mediates this relationship, utilizing data from the 2005 to 2010 NHANES.

## 2. Methods and Materials

### 2.1. Study Design

This analysis employed a cross-sectional approach utilizing data from the 2005 to 2010 cycles of the NHANES, an ongoing program implemented by the National Center for Health Statistics (NCHS) to evaluate the health and nutritional conditions of the U.S. population. NHANES applies a multistage, stratified, and probabilistic sampling strategy to produce estimates representative of the civilian, non-institutionalized population in the United States (CDC, 2022). Ethical approval for data collection was granted by the NCHS Research Ethics Review Board, and all participants provided written informed consent. The present study followed the STROBE (Strengthening the Reporting of Observational Studies in Epidemiology) guidelines for transparent reporting of observational research. (Public data are available at https://www.cdc.gov/nchs/nhanes/index.htm).

### 2.2. Population

**Figure 1:**
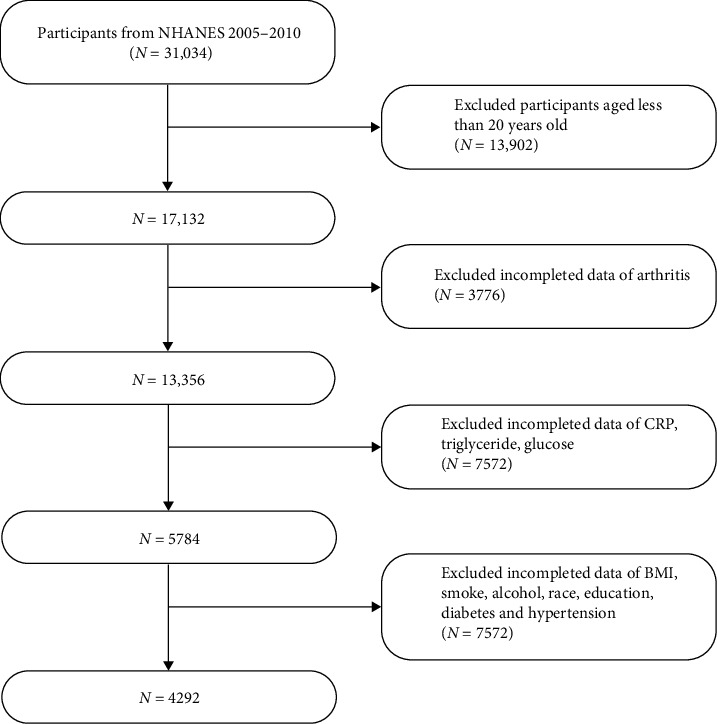
Flowchart of participant selection.

A total of 31,034 individuals were initially eligible. Participants younger than 20 years (*n* = 13,902) and those lacking data on RA diagnosis, CRP, triglycerides, or fasting glucose (*n* = 11,348) were excluded. Additional exclusions were made for missing data on key covariates, including BMI, race/ethnicity, educational attainment, smoking and alcohol use, diabetes status, and hypertension (*n* = 7572). After applying these criteria, the final analytic cohort consisted of 4292 adults with complete information on exposures, outcomes, and relevant covariates, enabling robust and comprehensive statistical analysis. A visual overview of participant inclusion is presented in Figure 1.

### 2.3. Exposure Variable

The main exposure in this study was the CTI, a composite measure capturing elements of systemic inflammation and insulin resistance. CTI was calculated using the following formula:

  $$\begin{array}{c} \text{CTI}=0.412\times \ln\left[\text{CRP(mg/L)}\right]+\ln\left[\text{triglyceride (mg/dL)}\times \text{fasting glucose (mg/dL)}/2\right]. \end{array}$$

As outlined in recent literature on metabolic-inflammatory risk indices [6]. This metric was evaluated in two forms: as a continuous variable to assess dose-response associations and as a categorical variable, stratified into quartiles (Q1–Q4) based on the weighted population distribution in the analytic dataset.

### 2.4. Outcome Variable

RA status was determined through self-reported information in the NHANES questionnaire. Participants were first asked whether a healthcare provider had ever diagnosed them with any form of arthritis. Those who responded affirmatively were then asked to specify the type. Individuals who selected “rheumatoid arthritis” were classified as RA cases, while those who did not report this subtype were categorized as non-RA. Although self-reported, this method of RA ascertainment has been validated and is widely utilized in large-scale epidemiological research [4, 15].

### 2.5. Covariates

Potential confounders were identified a priori based on their established associations with both CTI and RA. Demographic factors included sex, age, and race/ethnicity, categorized as non-Hispanic White, non-Hispanic Black, Mexican American, other Hispanic, and other/multiracial. Educational attainment was also included as a socioeconomic indicator. Lifestyle-related covariates comprised smoking status (never, former, current) and alcohol use (yes/no). Clinical variables included BMI, diabetes, and hypertension. BMI was calculated as weight in kilograms divided by height in meters squared (kg/m^2^) and classified according to World Health Organization (WHO) criteria: underweight (<18.5 kg/m^2^), normal weight (18.5–24.9 kg/m^2^), overweight (25.0–29.9 kg/m^2^), and obese (≥30.0 kg/m^2^). Diabetes status was defined by a combination of criteria: self-reported diagnosis, current insulin use, or meeting diagnostic thresholds such as hemoglobin A1c ≥6.5%, fasting plasma glucose ≥126 mg/dL, or a 2-h postload glucose level ≥200 mg/dL following an oral glucose tolerance test. Hypertension was defined as systolic blood pressure ≥140 mmHg, diastolic pressure ≥90 mmHg, self-reported diagnosis, or use of antihypertensive medications. All covariates were categorized as appropriate and incorporated into multivariable models to adjust for potential confounding. These variables have been consistently linked to systemic inflammation and RA susceptibility in prior research [11, 12].

### 2.6. Statistical Analysis

All statistical procedures were conducted using R software version 4.4.1 (R Core Team, 2023). To account for NHANES's complex, multistage sampling design and incorporate appropriate survey weights, the “survey” package in R was employed, allowing for nationally representative estimates. Descriptive analyses were performed for the full study population and stratified by CTI quartiles. Associations between CTI and RA were evaluated using logistic regression. Three models were constructed: Model 1 included no covariates. Model 2 adjusted for sex, age, and race/ethnicity. Model 3 further controlled for education, smoking status, alcohol consumption, diabetes, and hypertension. To examine potential nonlinear relationships, restricted cubic spline (RCS) models were applied. Subgroup analyses assessed effect modification by key demographic and clinical characteristics. To explore mediation, a counterfactual-based causal mediation framework was used to quantify the extent to which BMI mediated the CTI–RA relationship. Results were reported as odds ratios (ORs) with 95% confidence intervals (CIs). NHANES weights were incorporated throughout all analyses to ensure generalizability to the U.S. adult population. For missing data, cases with minimal missingness were excluded, while multiple imputation was applied for variables with higher proportions of missingness to preserve analytic robustness. All tests were two-tailed, and statistical significance was defined as *p*  < 0.05.

## 3. Results

### 3.1. Baseline Characteristics of Participants by CTI Quartiles

Baseline demographic and clinical characteristics of the 4,292 study participants are presented in Table 1. Statistically significant differences were observed across quartiles of the CTI distribution for the majority of variables (all *p* < 0.001). Individuals in the highest CTI group (Q4) were notably older, with a median age of 47 years, compared to 37 years in the lowest quartile (Q1). The proportion of male participants increased across CTI quartiles, rising from 45% in Q1 to 57% in Q3 and 52% in Q4. Markers of socioeconomic disadvantage were more prominent in the higher CTI categories, including lower educational attainment and reduced poverty-to-income ratios. Obesity showed a strong graded relationship with CTI: Only 8.7% of Q4 participants had a normal BMI, in contrast to 59% in Q1, while the prevalence of obesity increased from 8.0% in Q1 to 58% in Q4. Similar upward trends were seen for diabetes and hypertension, with diabetes prevalence rising sharply from 3.2% in Q1 to 32% in Q4. Smoking behavior also varied: current and former smoking rates were higher among those with elevated CTI, while the proportion of never smokers declined across quartiles. No consistent pattern was observed for alcohol use (*p*  =  0.400). Racial/ethnic distribution differed significantly, with a higher representation of Mexican Americans in the upper CTI quartiles. Importantly, the prevalence of RA increased steadily across CTI levels—from 1.8% in Q1 to 9.1% in Q4 (*p* < 0.001)—supporting a strong positive association between higher CTI values and RA risk (Table 1).

### 3.2. Association Between CTI and RA

Logistic regression analysis revealed a strong and statistically significant relationship between CTI and RA risk, whether CTI was assessed as a continuous variable or categorized into quartiles (Table 2). In the unadjusted model (Model 1), each one-unit rise in CTI was associated with a 79% increase in the odds of RA (OR = 1.79, 95% CI: 1.58–2.04, *p*  < 0.001). This association persisted after adjustment for age, sex, and race/ethnicity in Model 2 (OR = 1.67, 95% CI: 1.42–1.96, *p*  < 0.001) and remained robust in the fully adjusted model (Model 3), which also accounted for education level, smoking status, alcohol use, diabetes, and hypertension (OR = 1.45, 95% CI: 1.22–1.73, *p*  < 0.001).

When CTI was evaluated in quartiles, a clear dose–response pattern emerged. Compared with participants in the lowest quartile (Q1), those in higher quartiles had progressively increased odds of RA. In the unadjusted analysis, individuals in Q4 had over a fivefold greater likelihood of RA relative to Q1 (OR = 5.39, 95% CI: 3.13–9.29, *p*  < 0.001). Even after full adjustment in Model 3, Q4 participants maintained a significantly elevated risk (OR = 2.66, 95% CI: 1.41–5.01, *p* = 0.004). A significant linear trend across quartiles was observed in all models (p for trend < 0.001 in Models 1 and 2; *p* = 0.004 in Model 3), supporting the presence of a nonlinear dose–response association. Although Q2 was not statistically significant in Model 3 (*p* = 0.067), Q3 and Q4 retained significance, suggesting that the association between CTI and RA becomes more pronounced in the upper range of the index. Overall, these findings highlight CTI as an independent predictor of RA risk after adjusting for multiple demographic and clinical covariates (Table 2).

### 3.3. Nonlinear Relationship Between CTI and RA Risk

**Figure 2:**
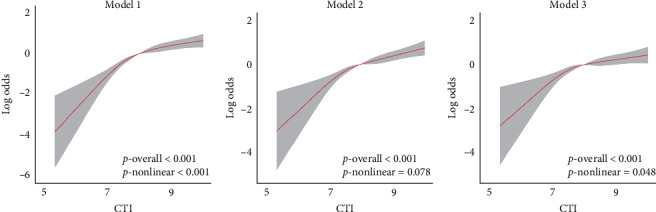
RCS analysis of the association between CTI and RA risk. Dose–response relationship between the CTI and RA risk based on RCS analysis. Subparts A, B, and C correspond to Model 1 (unadjusted), Model 2 (adjusted for sex, age, and race), and Model 3 (fully adjusted for sex, age, race, education, smoking, alcohol use, diabetes, and hypertension), respectively. The red line indicates the estimated log odds, and the shaded gray area denotes the 95% CI. (a) In Model 1, the relationship was significantly nonlinear (*p*  < 0.001 for both overall and nonlinear trend). (b) In Model 2, the overall trend remained significant (*p*  < 0.001), though the nonlinearity was attenuated (*p* = 0.078). (c) In the fully adjusted Model 3, the association remained significant overall (*p*  < 0.001), and a modest but statistically significant nonlinear pattern persisted (*p* = 0.048).

RCS analysis revealed a significant nonlinear relationship between CTI and the likelihood of developing RA (Figure 2). In the unadjusted model (Model 1), RA risk increased sharply once CTI exceeded approximately 7.0, with a highly significant overall association (*p*  < 0.001) and clear evidence of nonlinearity (*p*-nonlinear <0.001). This trend persisted in Model 2, which adjusted for age, sex, and race/ethnicity, showing a continued strong association (*p*  < 0.001), though the nonlinearity was less pronounced (*p*-nonlinear = 0.078). In the fully adjusted model (Model 3), which included additional covariates such as education, lifestyle, and clinical factors, the CTI remained significantly associated with RA risk (*p*  < 0.001), and the nonlinear pattern reemerged (*p*-nonlinear = 0.048).

### 3.4. Subgroup Analysis of the Association Between CTI and RA

Stratified analyses demonstrated that the positive association between CTI and RA was generally consistent across a wide range of demographic and clinical subgroups (Table 3). The relationship was statistically significant in both sexes, with no evidence of effect modification by gender (*p* for interaction = 0.760). Similarly, significant associations were observed among non-Hispanic White and Black participants. Although the association did not reach statistical significance among Mexican Americans and other minority groups—likely due to smaller subgroup sizes—no interaction by race/ethnicity was detected (*p* = 0.353). Educational level did not significantly influence the CTI–RA association (*p* = 0.642), with significant findings observed across most education categories. Lifestyle factors, including smoking status and alcohol consumption, also did not modify the association (*p* = 0.710 and 0.543, respectively), suggesting that behavioral variables exert limited influence on the observed relationship. The most notable effect modification emerged in the stratified analysis by diabetes status. Among non-diabetic individuals, CTI remained strongly associated with RA risk; however, in participants with diabetes, the association was attenuated and no longer statistically significant. This differential effect yielded a significant interaction term (*p* = 0.036), potentially reflecting the confounding influence of advanced metabolic dysregulation. In contrast, hypertension status did not significantly alter the association (*p* = 0.695). Collectively, these findings reinforce the robustness of the CTI–RA association across most population strata and highlight its potential value as a predictive biomarker, particularly in individuals without overt metabolic disease.

### 3.5. Mediation Effect of BMI in the Association Between CTI and RA

To further explore the potential mediating role of BMI in the relationship between CTI and RA, we conducted a causal mediation analysis. Logistic regression results demonstrated a strong, positive association between CTI and BMI, with each unit increase in CTI corresponding to a 3.2 kg/m^2^ increase in BMI after full adjustment (*p*  < 0.001, Table S1). BMI increased progressively across CTI quartiles, reinforcing the linear relationship between systemic metabolic-inflammatory burden and adiposity. In addition, higher BMI was independently associated with increased RA risk when treated as a continuous variable, with each unit increase in BMI corresponding to a 5%–6% increase in the odds of RA (*p*  < 0.001, Table S2). However, when BMI was analyzed categorically, no significant associations were observed, suggesting that BMI is a more reliable predictor of RA risk when considered as a continuous measure.

**Figure 3:**
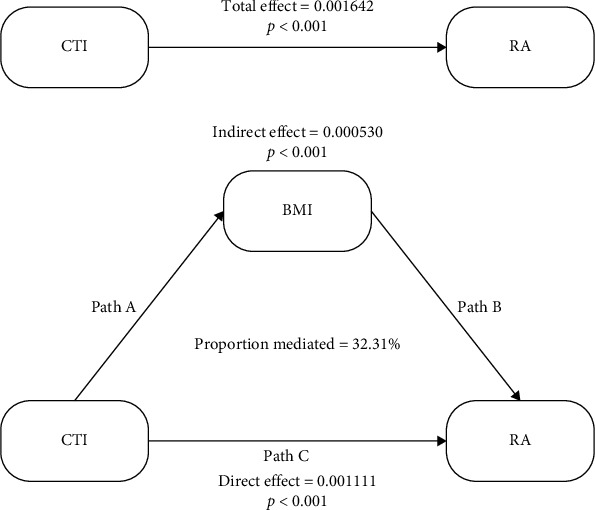
Causal mediation analysis of the relationship between CTI and RA mediated by BMI. Mediation model illustrating the indirect effect of BMI in the association between the CTI and RA. CTI was modeled as the independent variable, BMI as the mediator, and RA as the outcome. Path A represents the effect of CTI on BMI, Path B represents the effect of BMI on RA, and Path C indicates the direct effect of CTI on RA after accounting for BMI. The total effect of CTI on RA was statistically significant (coefficient = 0.001642, *p*  < 0.001), with an indirect effect via BMI of 0.000530 (*p*  < 0.001) and a direct effect of 0.001111 (*p*  < 0.001). The mediation analysis revealed that BMI accounted for 32.31% of the total effect, suggesting partial mediation.

To examine whether BMI mediates the association between CTI and RA, we conducted a causal mediation analysis, with CTI as the independent variable, BMI as the mediator, and RA as the outcome (Figure 3). The mediation analysis revealed that BMI significantly mediated the relationship between CTI and RA. The indirect effect of BMI was statistically significant (coefficient = 0.000530, 95% CI: 0.000149–0.001546, *p*  < 0.001), accounting for 32.31% of the total effect. Importantly, the direct effect of CTI on RA remained significant, even after adjusting for the mediating role of BMI (coefficient = 0.001111, 95% CI: 0.000502–0.001767, *p*  < 0.001), and the total effect was also significant (coefficient = 0.001642, 95% CI: 0.000677–0.003030, *p*  < 0.001). These findings indicate that BMI partially mediates the association between systemic inflammation and metabolic dysfunction, as represented by CTI, and the risk of developing RA. This underscores BMI as a critical intermediary in the pathway linking inflammation-related metabolic dysregulation to autoimmune disease (Table S3).

## 4. Discussion

Our study demonstrates that the CTI is independently and nonlinearly associated with RA risk in a nationally representative U.S. adult population. Higher CTI values were consistently related to increased RA risk, with the association becoming more pronounced at the upper end of the CTI distribution. Importantly, we identified BMI as a partial mediator of this relationship, explaining nearly one-third of the total effect. These results highlight CTI as a novel composite biomarker that integrates inflammatory and metabolic stress and provide new evidence that adiposity-related pathways may contribute to RA pathogenesis.

Our findings align with a growing body of literature highlighting the interplay between systemic inflammation, metabolic dysregulation, and the development of RA. Elevated CRP levels have long been associated with increased RA incidence and disease activity, underscoring the role of inflammatory biomarkers in both early detection and disease monitoring [16, 17]. In parallel, the triglyceride–glucose (TyG) index—commonly used as a proxy for insulin resistance—has also been linked to arthritis risk, particularly in younger or metabolically active populations [18]. Consistently, a recent large NHANES-based study reported that higher neutrophil percentage-to-albumin ratio was positively associated with RA but not with Osteoarthritis, further supporting the relevance of systemic inflammation in RA susceptibility [19]. However, our study extends these observations by employing the CTI, a composite measure that integrates CRP with the TyG index to reflect both inflammatory and metabolic burden more comprehensively. To our knowledge, this is the first large-scale, population-based study to establish a direct relationship between CTI and RA risk. While previous research has applied CTI in the context of cardiovascular events [5], cancer mortality [20], and depressive symptoms [6], its relevance to autoimmune conditions such as RA had not been examined prior to this work. Unlike earlier studies that assessed CRP or TyG in isolation, our analysis offers novel insights by evaluating CTI as a unified marker and examining its underlying mechanism of action. Importantly, our mediation analysis adds a new dimension to existing knowledge by identifying BMI as a significant mediator in the CTI–RA association. Although BMI has been previously recognized as a risk factor for RA onset and as a modifier of treatment outcomes [12, 21], few studies have quantified its mediating effect in a causal framework. Furthermore, our use of RCS modeling revealed a nonlinear dose–response pattern, suggesting a threshold beyond which RA risk escalates more steeply with rising CTI levels. This nonlinear effect may inform future strategies for early risk stratification and targeted intervention in populations with elevated metabolic-inflammatory profiles.

A key strength of this study lies in its rigorous analytical framework, which incorporated advanced statistical methods to yield a more nuanced understanding of the CTI–RA relationship. Unlike previous investigations that relied on linear modeling assumptions, we employed RCS analysis to explore potential nonlinearity. This approach revealed a significant nonlinear association, highlighting a threshold effect whereby RA risk increases disproportionately at higher CTI values—an insight that would likely be missed using conventional linear regression [22]. In addition, our use of causal mediation analysis enabled us to disentangle the role of BMI as a mediator. Although prior research has implicated BMI in RA development [11], few studies have quantified its indirect contribution within a causal framework. Our results demonstrated that BMI accounted for approximately 32.3% of the total effect linking CTI to RA risk. By utilizing a counterfactual-based approach with bootstrapped CIs, our mediation analysis offers greater robustness and interpretability than traditional methods such as the Sobel test or simple path models—particularly in observational settings where confounding and nonlinearity must be carefully addressed [23].

Additionally, we treated CTI both as a continuous and a categorical variable (quartiles), enabling us to assess not only linear trends but also dose–response relationships. This dual analytic strategy enhanced the interpretability and clinical applicability of our results, particularly in risk stratification. Importantly, we used a large, nationally representative dataset (NHANES 2005–2010), which increases the generalizability of our findings to U.S. adults and strengthens external validity [24]. Subgroup analyses by sex, race, smoking, and metabolic status further confirmed the robustness of the CTI–RA association, with interaction testing revealing stronger associations in non-diabetic participants—an observation not previously reported. Collectively, these methodological innovations provide novel insights and a solid framework for future longitudinal validation and mechanistic exploration.

The relationship between elevated CTI and increased RA risk—partially mediated by BMI—likely reflects the convergence of multiple interconnected biological pathways involving systemic inflammation, insulin resistance, and adiposity-driven immune dysregulation. CRP, a core component of CTI, is a well-known acute-phase reactant and marker of systemic inflammation. Elevated CRP levels contribute to synovial inflammation and joint damage in RA through complement activation and the upregulation of pro-inflammatory cytokines such as interleukin-6 and tumor necrosis factor-α [25, 26]. In parallel, elevated triglycerides and fasting glucose—reflective of underlying insulin resistance—can perpetuate chronic low-grade inflammation through oxidative stress, impaired lipid handling, and macrophage polarization toward the pro-inflammatory M1 phenotype [27, 28]. Insulin resistance may also directly influence immune function by promoting hyperinsulinemia and hyperglycemia, which have been shown to dysregulate T-cell responses, favoring Th17 differentiation and reducing regulatory T-cell activity—key mechanisms implicated in RA pathogenesis [29, 30]. Adipose tissue further amplifies this inflammatory milieu. In obese individuals, both adipocytes and infiltrating immune cells secrete adipokines such as leptin, resistin, and visfatin, which enhance inflammatory signaling and promote autoimmune activation [9, 31]. Leptin, in particular, has been shown to favor Th1 and Th17 responses while suppressing anti-inflammatory regulatory pathways, thereby contributing to RA onset and progression [32]. Together, these mechanisms illustrate how CTI captures the integrated burden of metabolic inflammation, adipokine imbalance, and immune activation. BMI, as both a mediator and synergistic contributor, magnifies these effects, reinforcing the value of CTI as a comprehensive biomarker for identifying individuals at heightened risk for RA through overlapping metabolic and immunological pathways.

While this study offers important insights—benefiting from a large, nationally representative sample, rigorous covariate adjustment, and advanced statistical methodologies—several limitations warrant consideration. First, the cross-sectional nature of NHANES limits causal inference. Although our mediation analysis provides preliminary evidence of potential pathways linking CTI, BMI, and RA, the absence of temporal sequencing prevents definitive conclusions about causality [33]. Specifically, NHANES data do not allow us to evaluate the temporal evolution between CTI elevation and the onset of RA symptoms, and therefore we cannot establish whether increased CTI precedes RA development or reflects existing disease activity. Longitudinal studies will be necessary to clarify this temporal relationship. Second, RA status was determined through self-reported physician diagnosis. Although this measure has been validated in previous NHANES analyses, the potential for misclassification remains, which could lead to non-differential bias and attenuation of observed associations [15]. Moreover, self-reported diagnosis cannot capture disease activity or severity and lacks the precision of quantitative biomarkers such as CRP levels or standardized indices of insulin resistance, which have been highlighted in prior studies as important for mechanistic interpretation [25, 34]. In addition, our dataset did not provide information on RA disease course, such as exacerbations, remissions, or flares. However, prior studies have shown that obesity is associated with higher disease activity and lower likelihood of remission in RA [12, 13], suggesting that BMI and related metabolic burden may influence RA phenotypes over time. Third, despite extensive adjustment for known confounders, residual confounding cannot be ruled out. Unmeasured factors such as physical activity, psychological stress, dietary quality, and genetic predisposition may influence both CTI and RA risk. Fourth, while BMI served as a proxy for adiposity in our mediation analysis, it represents a limited and indirect measure. Metrics such as visceral fat content, waist circumference, or waist-to-hip ratio may better reflect the inflammatory potential of adipose tissue [35]. Additionally, the distinction between general obesity and adiposity is critical: prior evidence shows that central adiposity exerts stronger pro-inflammatory effects than BMI-defined obesity alone [36]. The existence of the “metabolically healthy obesity (MHO)” phenotype further complicates interpretation, as some obese individuals may maintain preserved insulin sensitivity and relatively low systemic inflammation [29, 37, 38], which our analysis could not disentangle. Finally, our study did not directly evaluate metabolic syndrome, which has been shown to be associated with increased RA risk and worse outcomes [34, 39]. Given that CTI integrates markers of inflammation and insulin resistance, it may partly overlap with the pathophysiological features of metabolic syndrome. Future research incorporating standardized definitions of metabolic syndrome will help clarify its interplay with CTI and RA.

Future longitudinal studies are essential to validate the hypothesized causal pathways and to explore the predictive value of CTI for RA onset and disease progression. Prospective research incorporating clinically confirmed RA diagnoses, imaging modalities, and detailed body composition assessments will help clarify the biological mechanisms underlying our findings. Integrating refined adiposity metrics with inflammatory biomarkers may allow better discrimination of risk subgroups. Additionally, incorporating genomic data and evaluating gene–environment interactions may enhance risk stratification and shed light on interindividual variability in RA susceptibility. Finally, assessing whether CTI can serve as a predictive marker for RA development or treatment response in early-phase cohorts may hold promise for clinical translation.

## 5. Conclusions

This study highlights CTI as a promising biomarker reflecting the interplay between metabolic dysfunction and systemic inflammation in relation to RA risk. It emphasizes the importance of considering obesity-related pathways in autoimmune disease development. Future research should focus on longitudinal studies to establish causality, incorporate more precise measures of adiposity, and explore molecular mechanisms using omics approaches. An integrative framework combining metabolic, inflammatory, and lifestyle factors may enhance early identification and prevention strategies for RA.

## Figures and Tables

**Table 1:** Characteristics of included participants.

Characteristic	Overall	CTI quartiles				
	Overall *N* = 4292^a^	Q1 *N* = 944^a^	Q2 *N* = 1029^a^	Q3 *N* = 1116^a^	Q4 *N* = 1203^a^	*p* value^b^
Sex						<0.001
Female	2025 (48%)	509 (55%)	462 (45%)	485 (43%)	569 (48%)	—
Male	2267 (52%)	435 (45%)	567 (55%)	631 (57%)	634 (52%)	—
Age (years)	44 (32, 55)	37 (27, 48)	44 (32, 55)	46 (34, 59)	47 (36, 58)	<0.001
Age quartiles						<0.001
20–40 years	1614 (41%)	525 (56%)	393 (40%)	362 (36%)	334 (32%)	—
40–60 years	1516 (41%)	290 (35%)	373 (43%)	376 (41%)	477 (46%)	—
60+ years	1162 (18%)	129 (9.7%)	263 (17%)	378 (23%)	392 (23%)	—
Race						<0.001
Non-Hispanic White	1970 (69%)	450 (69%)	483 (69%)	493 (68%)	544 (69%)	—
Mexican American	919 (10%)	145 (7.1%)	202 (9.6%)	251 (11%)	321 (13%)	—
Non-Hispanic Black	815 (11%)	213 (12%)	209 (11%)	218 (11%)	175 (8.9%)	—
Other Hispanic	418 (5.1%)	81 (4.3%)	92 (4.6%)	111 (5.7%)	134 (5.6%)	—
Other/multiracial	170 (5.3%)	55 (7.2%)	43 (5.6%)	43 (5.1%)	29 (3.3%)	—
Education						<0.001
Less than 9th grade	511 (6.1%)	59 (3.2%)	103 (5.3%)	153 (6.9%)	196 (8.9%)	—
9–11th grade	671 (11%)	122 (8.5%)	157 (12%)	173 (10%)	219 (14%)	—
High school grad/GED	1004 (24%)	182 (18%)	240 (23%)	297 (29%)	285 (25%)	—
Some college or AA degree	1205 (30%)	290 (31%)	285 (28%)	292 (29%)	338 (33%)	—
College graduate or above	901 (29%)	291 (40%)	244 (32%)	201 (25%)	165 (19%)	—
PIR	3.26 (1.68, 5.00)	3.43 (1.89, 5.00)	3.63 (1.90, 5.00)	3.13 (1.62, 5.00)	2.77 (1.40, 4.84)	<0.001
BMI quartiles (kg/m^2^)						<0.001
Underweight	61 (1.6%)	43 (4.8%)	11 (0.9%)	3 (0.3%)	4 (0.2%)	—
Normal	1239 (31%)	540 (59%)	338 (38%)	245 (20%)	116 (8.7%)	—
Overweight	1529 (35%)	280 (28%)	430 (41%)	425 (38%)	394 (33%)	—
Obese	1463 (32%)	81 (8.0%)	250 (21%)	443 (42%)	689 (58%)	—
Smoke						<0.001
Never smoker	2358 (55%)	609 (64%)	567 (55%)	603 (53%)	579 (48%)	—
Former smoker	1029 (24%)	172 (19%)	231 (21%)	283 (25%)	343 (30%)	—
Current smoker	905 (21%)	163 (17%)	231 (23%)	230 (21%)	281 (23%)	—
Alcohol						0.400
Drinker	3768 (90%)	832 (90%)	912 (90%)	986 (91%)	1038 (88%)	—
Nondrinker	524 (10%)	112 (10%)	117 (9.8%)	130 (8.8%)	165 (12%)	—
Diabetes						<0.001
No	3452 (86%)	901 (97%)	918 (92%)	914 (86%)	719 (68%)	—
Yes	840 (14%)	43 (3.2%)	111 (7.5%)	202 (14%)	484 (32%)	—
Hypertension						<0.001
No	3008 (75%)	816 (90%)	784 (81%)	731 (70%)	677 (61%)	—
Yes	1284 (25%)	128 (10%)	245 (19%)	385 (30%)	526 (39%)	—
CTI	7.88 (7.24, 8.53)	6.78 (6.44, 7.03)	7.58 (7.41, 7.72)	8.19 (8.04, 8.35)	8.95 (8.72, 9.32)	<0.001
Arthritis	329 (5.7%)	22 (1.8%)	62 (4.4%)	101 (7.5%)	144 (9.1%)	<0.001
						

*Note:* For analytic purposes, the continuous variable CTI was categorized into four groups according to its quartile values, designated as Q1 (lowest quartile), Q2, Q3, and Q4 (highest quartile).

Abbreviations: BMI, body mass index; CTI, C-reactive protein–triglyceride–glucose index; PIR, poverty-to-income ratio.

^a^ *n* (unweighted) (%); median (Q1, Q3).

^b^Pearson's *X*^2^: Rao and Scott adjustment; design-based Kruskal–Wallis test.

**Table 2:** The associations between CTI and RA.

Characteristic	Model 1		Model 2		Model 3	
	OR (95% CI)	*p* value	OR (95% CI)	*p* value	OR (95% CI)	*p* value
CTI (continuous)	1.79 (1.58, 2.04)	<0.001	1.67 (1.42, 1.96)	<0.001	1.45 (1.22, 1.73)	<0.001
CTI quartiles						
Q1	—	—	—	—	—	—
Q2	2.46 (1.35, 4.48)	0.004	1.88 (1.03, 3.44)	0.040	1.74 (0.96, 3.16)	0.067
Q3	4.31 (2.23, 8.37)	<0.001	3.02 (1.48, 6.13)	0.003	2.46 (1.18, 5.13)	0.018
Q4	5.39 (3.13, 9.29)	<0.001	3.76 (2.07, 6.84)	<0.001	2.66 (1.41, 5.01)	0.004
*p* for trend	<0.001		<0.001		0.004	

*Note:* For analytic purposes, the continuous variable CTI was categorized into four groups according to its quartile values, designated as Q1 (lowest quartile), Q2, Q3, and Q4 (highest quartile). Model 1: unadjusted model. Model 2: adjusted for sex, age and race. Model 3: adjusted for sex, age, race, education, smoke status, alcohol consumption, diabetes, and hypertension.

Abbreviations: CI, confidence interval; CTI, C-reactive protein–triglyceride–glucose index; OR, odds ratio; RA, rheumatoid arthritis.

**Table 3:** Subgroup analysis of the associations between CTI and RA.

Characteristic	OR (95% CI)	*p* value	*p* for interaction
Sex			0.760
Male	1.44 (1.08, 1.92)	0.014	—
Female	1.47 (1.14, 1.88)	0.004	—
Race			0.353
Non-Hispanic White	1.52 (1.20, 1.91)	<0.001	—
Non-Hispanic Black	1.50 (1.17, 1.93)	0.003	—
Mexican American	1.17 (0.76, 1.82)	0.5	—
Other Hispanic	1.45 (0.77, 2.74)	0.2	—
Other/multiracial	1.32 (0.59, 2.95)	0.5	—
Education			0.642
Less than 9th grade	1.06 (0.68, 1.65)	0.8	—
9–11th grade	1.72 (1.15, 2.58)	0.010	—
High school grad/GED	1.50 (1.04, 2.15)	0.032	—
Some college or AA degree	1.39 (1.02, 1.89)	0.036	—
College graduate or above	1.96 (1.14, 3.37)	0.017	—
Smoke			0.710
Current smoker	1.80 (1.34, 2.43)	<0.001	—
Former smoker	1.50 (0.97, 2.32)	0.069	—
Never smoker	1.33 (1.01, 1.75)	0.045	—
Alcohol			0.543
Drinker	1.47 (1.22, 1.79)	<0.001	—
Non-drinker	1.20 (0.71, 2.04)	0.5	—
Diabetes			0.036
Diabetes	1.19 (0.92, 1.53)	0.2	—
Nondiabetes	1.64 (1.29, 2.09)	<0.001	—
Hypertension			0.695
Hypertension	1.38 (1.11, 1.72)	0.005	—
Nonhypertension	1.50 (1.11, 2.01)	0.009	—

Abbreviations: CI, confidence interval; CTI, C-reactive protein–triglyceride–glucose index; OR, odds ratio; RA, rheumatoid arthritis.

## Data Availability

The data that support the findings of this study are available in NHANES at https://www.cdc.gov/nchs/nhanes/index.html. These data were derived from the following resources available in the public domain: https://www.cdc.gov/nchs/nhanes/index.html.

## Nomenclature

BMI: Body mass index
CI: Confidence interval
CRP: C-reactive protein
CTI: C-reactive protein–triglyceride–glucose index
NCHS: National Center for Health Statistics
NHANES: National Health and Nutrition Examination Survey
OR: Odds ratios
RA: Rheumatoid arthritis
RCS: Restricted cubic spline
TyG: Triglyceride–glucose index
WHO: World Health Organization
MHO: Metabolically healthy obesity.

## Supporting Information

Supporting Information Table S1. The associations between CTI and BMI. Table S2. The associations between BMI and RA. Table S3. The mediation effect of BMI score for the associations between CTI and RA.
