# Supplementary material for: Nonlinear Association Between the C-Reactive Protein–Triglyceride–Glucose Index and Rheumatoid Arthritis Risk: The Mediating Role of Body Mass Index
Source: Mediators Inflamm. 2025 Nov 10;2025:8729780. doi: 10.1155/mi/8729780 (PMC12623103; doi:10.1155/mi/8729780)
Supplement: Supporting Information — Table S1. The associations between CTI and BMI. Table S2. The associations between BMI and RA. Table S3. The mediation effect of BMI score for the associations between CTI and RA. [file 8729780.f1.docx]

**Supplementary Table 1 The associations between CTI and BMI**

|  | **Model 1** | | **Model 2** | | **Model 3** | |
| --- | --- | --- | --- | --- | --- | --- |
|  | **β (95% CI)** | **p value** | **β (95% CI)** | **p value** | **β (95% CI)** | **p value** |
| **CTI (continuous)** | 3.3 (3.1, 3.5) | <0.001 | 3.4 (3.2, 3.6) | <0.001 | 3.2 (2.9, 3.4) | <0.001 |
| **CTI quartiles** |  |  |  |  |  |  |
| Q1 | — |  | — |  | — |  |
| Q2 | 2.7 (2.2, 3.1) | <0.001 | 2.8 (2.4, 3.2) | <0.001 | 2.9 (2.5, 3.3) | <0.001 |
| Q3 | 5.7 (5.3, 6.1) | <0.001 | 5.9 (5.4, 6.4) | <0.001 | 5.7 (5.1, 6.2) | <0.001 |
| Q4 | 8.3 (7.7, 8.8) | <0.001 | 8.4 (7.9, 9.0) | <0.001 | 7.9 (7.3, 8.4) | <0.001 |
| CTI = C-reactive protein-triglyceride-glucose index, CI = Confidence Interval, BMI = Body mass index. For analytic purposes, the continuous variable CTI was categorized into four groups according to its quartile values, designated as Q1 (lowest quartile), Q2, Q3, and Q4 (highest quartile).  Model 1: unadjusted model.  Model 2: adjusted for sex, age and race.  Model 3: adjusted for sex, age, race, education, smoke status, alcohol consumption, diabetes and hypertension. | | | | | | |

**Supplementary Table 2 The associations between BMI and RA**

|  | **Model 1** | | **Model 2** | | **Model 3** | |
| --- | --- | --- | --- | --- | --- | --- |
|  | **OR (95% CI)** | **p value** | **OR (95% CI)** | **p value** | **OR (95% CI)** | **p value** |
| **BMI (continuous)** | 1.06 (1.04, 1.08) | <0.001 | 1.06 (1.04, 1.09) | <0.001 | 1.05 (1.03, 1.08) | <0.001 |
| **BMI quartiles (kg/m^2^)** |  |  |  |  |  |  |
| Underweight | — |  | — |  | — |  |
| Normal | 0.76 (0.15, 3.81) | 0.700 | 0.67 (0.12, 3.80) | 0.600 | 0.81 (0.16, 4.18) | 0.800 |
| Overweight | 1.00 (0.21, 4.82) | >0.900 | 0.80 (0.15, 4.32) | 0.800 | 0.91 (0.19, 4.44) | >0.900 |
| Obese | 2.13 (0.45, 10.00) | 0.300 | 1.81 (0.34, 9.53) | 0.500 | 1.81 (0.39, 8.34) | 0.400 |
| RA = Rheumatoid Arthritis, OR = Odds Ratio, CI = Confidence Interval, BMI = Body mass index.  Model 1: unadjusted model.  Model 2: adjusted for sex, age and race.  Model 3: adjusted for sex, age, race, education, smoke status, alcohol consumption, diabetes and hypertension. | | | | | | |

**Supplementary Table 3 The mediation effect of BMI score for the associations between CTI and RA**

| **Effect Decomposition** | **Coefficient (95% CI)** | **p value** | **Proportion mediated, %** |
| --- | --- | --- | --- |
| Indirect Effect | 0.000530 (0.000149, 0.001546) | <0.001 | 32.31% |
| Direct Effect | 0.001111 (0.000502, 0.001767) | <0.001 | — |
| Total Effect | 0.001642 (0.000677, 0.003030) | <0.001 | — |
| CTI = C-reactive protein-triglyceride-glucose index，RA = Rheumatoid Arthritis, BMI = Body mass index, CI = Confidence Interval | | | |
